# Supplementary material for: Monoclonal Antibodies Specific for SARS-CoV-2 Spike Protein Suitable for Multiple Applications for Current Variants of Concern
Source: Viruses. 2022 Dec 31;15(1):139. doi: 10.3390/v15010139 (PMC9863740; doi:10.3390/v15010139)
Supplement: Supplementary file 1 [file viruses-15-00139-s001.zip › viruses-2019381-supplementary.pdf]

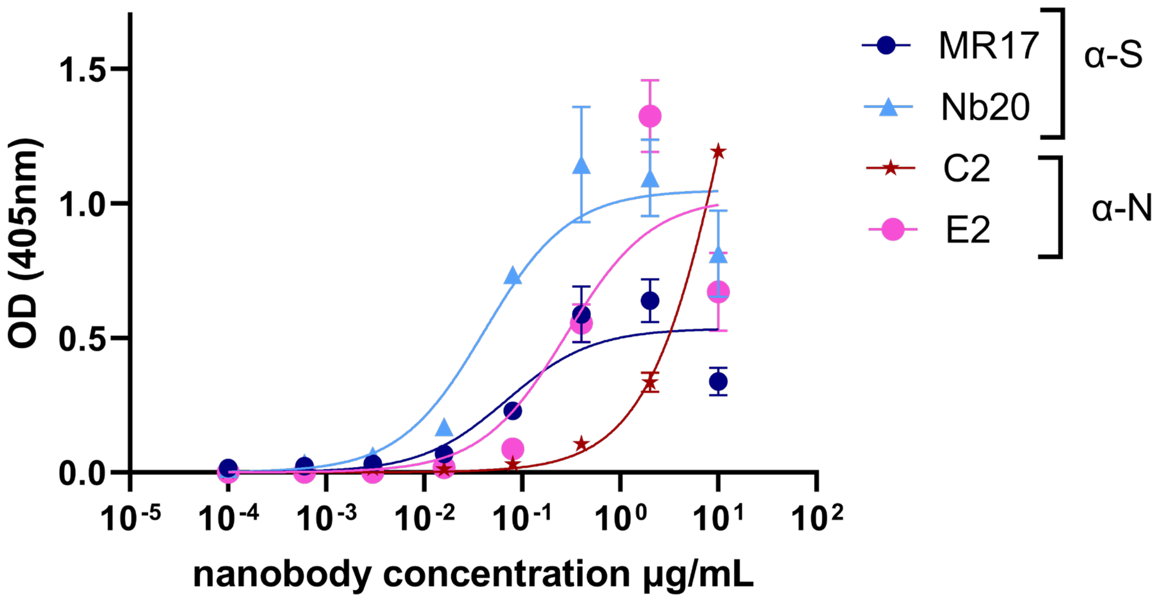

Supplementary Figure S1. ELISA titrations using serial dilutions of the indicated purified nanobodies on Vero E6 cells infected with SARS-CoV-2_{QLD02} and fixed in 80% acetone. The nanobodies were directed to spike (MR17, Nb20) or nucleocapsid (C2, E2) proteins.

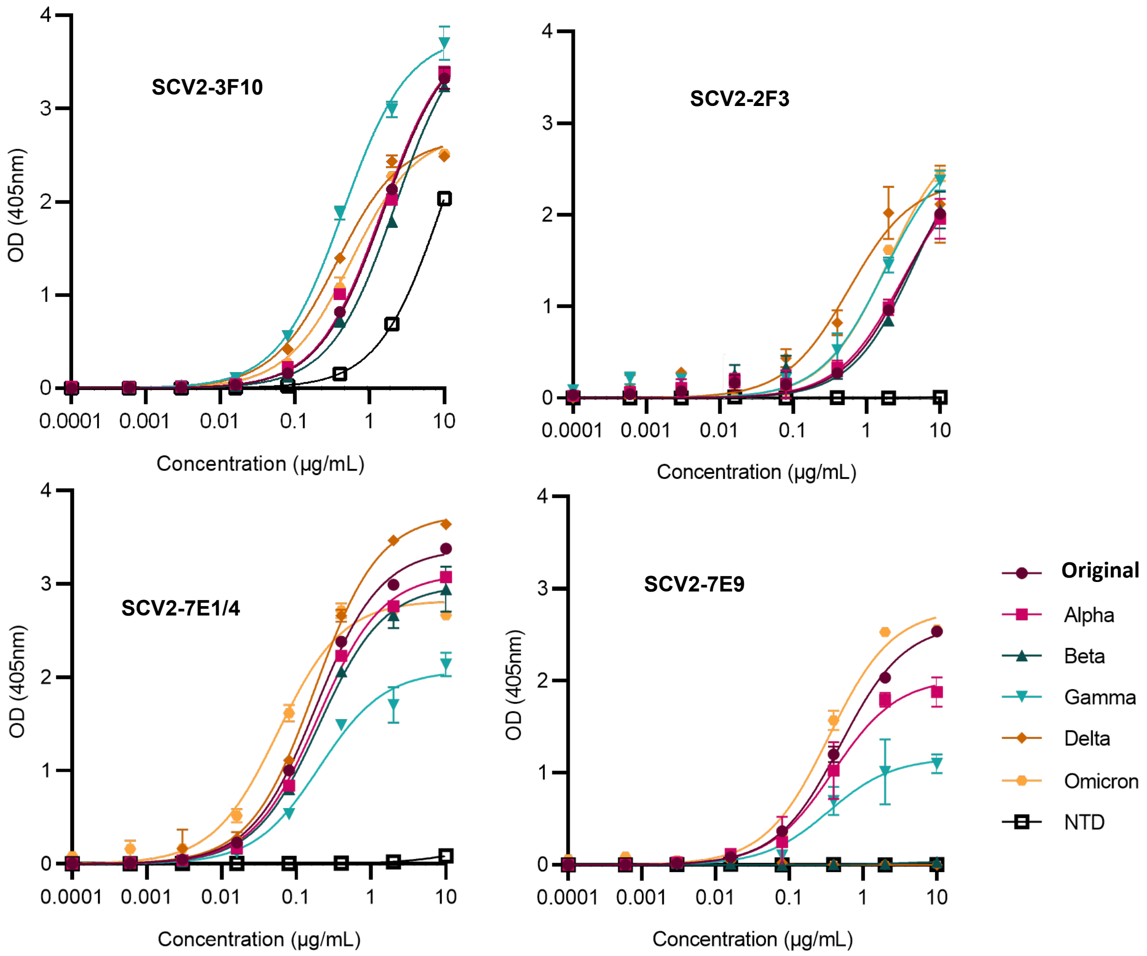

Supplementary Figure S2. ELISA titrations (summarised in Table 1) using serial dilutions of the indicated purified mAbs, and as antigens (i) recombinant HexaPro spike proteins with a mutated furin cleavage site from the indicated strains of concern, and (ii) recombinant N-terminal domain (NTD) of Wu-Hu-1 fused to human Fc.

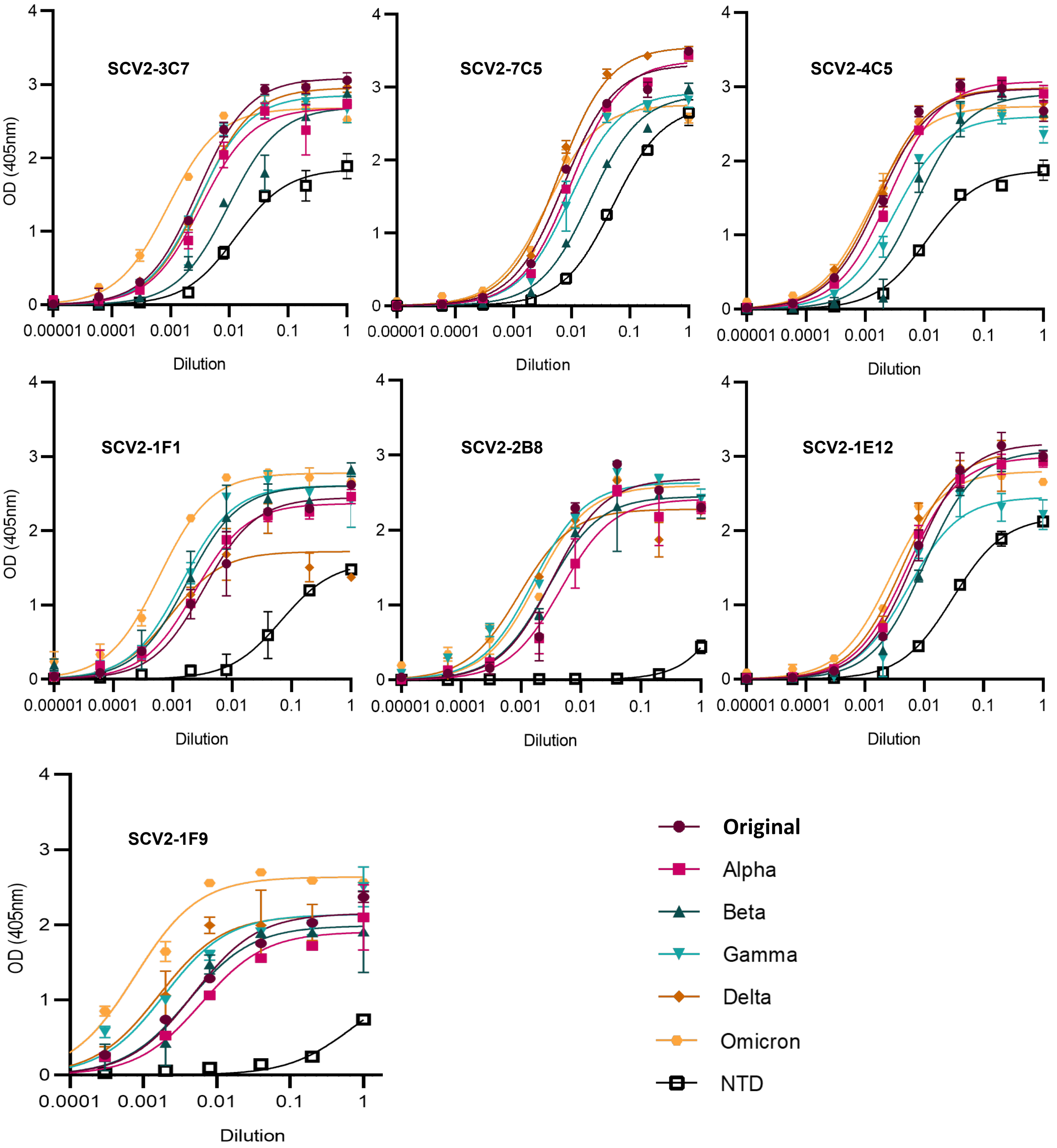

Supplementary Figure S3. As for Supplementary Fig. 2 but using the indicated dilutions of mAb tissue culture supernatants (1 represents neat supernatant).

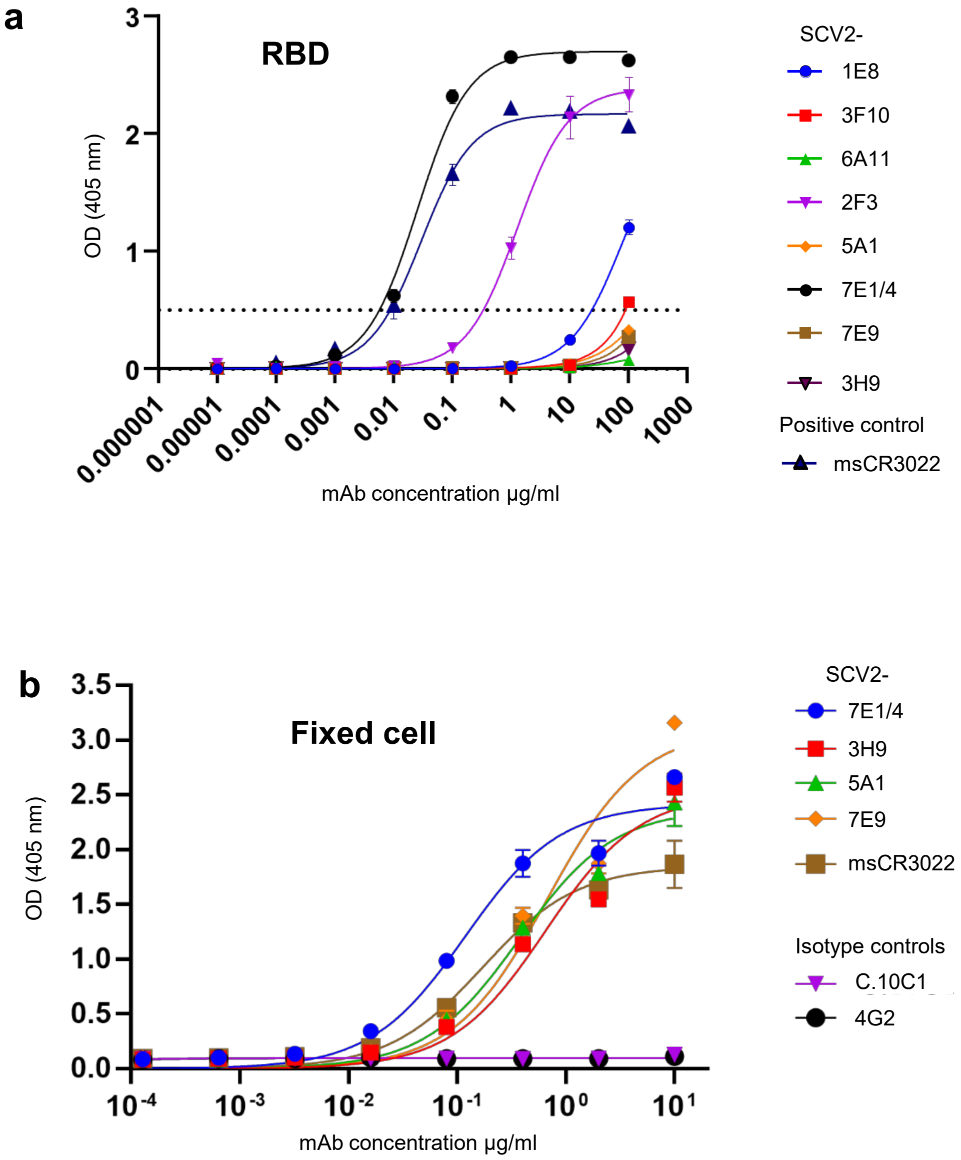

Supplementary Figure S4. (a) ELISAs using recombinant RBD (Wu-Hu-1) fused to human Fc as antigen (coating with 2 µg/ml), and serial dilutions of the indicated mAbs. Data is summarized in Table 1. msCR3022 is a positive control recombinant mouse anti-SARS-CoV-2 spike antibody (Rawle et al., 2021). (b) ELISAs using Vero E6 cells infected with SARS-CoV-2_{QLD02} and fixed in 80% acetone.

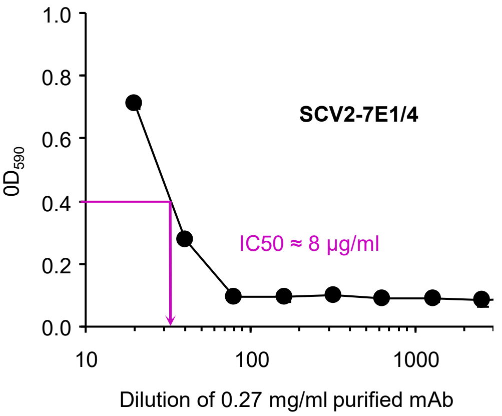

Supplementary Figure S5. Neutralisation assay as for Fig. 5. Note low concentration of purified mAb. Data from single replicates only.
